# Supplementary material for: Identification of photoperiod-induced specific miRNAs in the adrenal glands of Sunite sheep (Ovis aries)
Source: Front Vet Sci. 2022 Jul 22;9:888207. doi: 10.3389/fvets.2022.888207 (PMC9354845; doi:10.3389/fvets.2022.888207)
Supplement: Supplementary file 1 [file Data_Sheet_1.ZIP › Supplementary file/miRDeep2 file.zip!/miRDeep2 file/Novel_Structure/LP42A2/Novel_180.pdf]

Provisional ID	:	NC_019480.2_34023
Score total	:	13.3
Score for star read(s)	:	3.9
Score for read counts	:	5.7
Score for mfe	:	2
Score for randfold	:	1.6
Score for cons. seed	:	
Total read count	:	23
Mature read count	:	20
Loop read count	:	0
Star read count	:	3

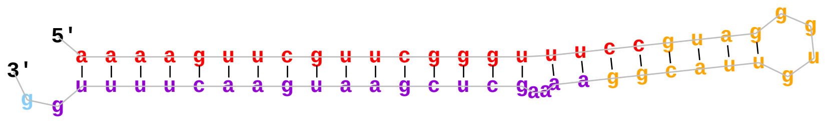

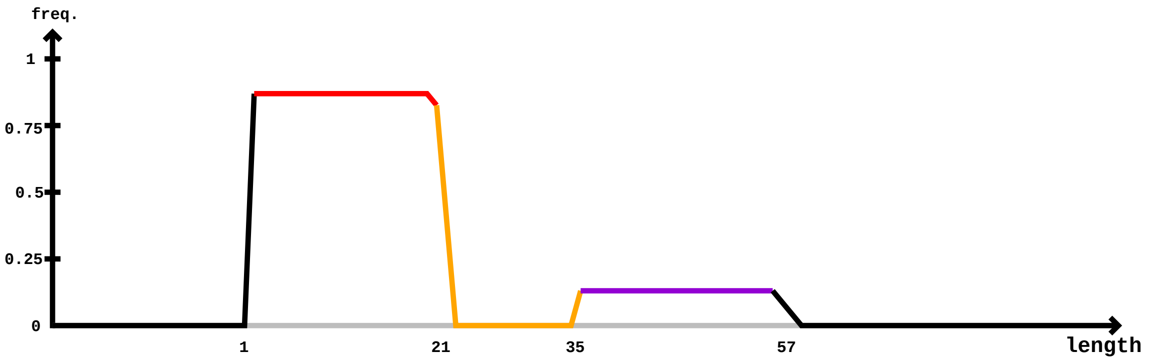

Mature Star

```
5'- cgauauacugaguuagcccaaaaguucguucggguuuccguaggguguuacggaaaagcucgaaugaacuuuugggccaacccaauacagagaacuucaggugcacaac  -3'   obs
    cgauauacugaguuagcccaaaaguucguucggguuuccguaggguguuacggaaaagcucgaaugaacuuuugggccaacccaauacagagaacuucaggugcacaac         exp
    ........((.(((.((((((((((((((((((((((((((((....))))))))..)))))))))))))))))))).))).)).........................    reads     mm     sample
    ...................aaaaguucguucggguuuc.......................................................................    1         0      seq
    ...................aaaaguucguucggguuuUc......................................................................    19        1      seq
    .....................................................aaaagcucAaaugaacuuuug...................................    3         1      seq
```
